# A Saliva-Based Serological and Behavioral Analysis of SARS-CoV-2 Antibody Prevalence in Howard County, Maryland

**DOI:** 10.1128/spectrum.02765-22

**Published:** 2023-06-08

**Authors:** Alan C. Brown, Phillip T. Koshute, Hannah P. Cowley, Michael S. Robinette, Sarah R. Gravelyn, Shraddha V. Patel, Eunice Y. Ju, Carolyn T. Frommer, Alexander E. Zambidis, Eric J. Schneider, Martina Y. Zhao, Benny K. Mugo, William Clarke, Kate Kruczynski, Nora Pisanic, Christopher D. Heaney, Teresa A. Colella

**Affiliations:** a Johns Hopkins University Applied Physics Laboratory, Laurel, Maryland, USA; b Johns Hopkins Medicine, Baltimore, Maryland, USA; c Johns Hopkins Environmental Health Microbiology and Immunology Laboratory (JH-EHMIL), Bloomberg School of Public Health, Baltimore, Maryland, USA; Quest Diagnostics Nichols Institute

**Keywords:** BNT162b2, Howard County, Maryland, SARS-CoV-2, antibody decay rate, community health study, mRNA-1273, oral fluid SARS-CoV-2 IgG assay, seroprevalence

## Abstract

The objective of the study was to estimate severe acute respiratory syndrome coronavirus 2 (SARS-CoV-2) seroprevalence in the Howard County, Maryland, general population and demographic subpopulations attributable to natural infection or coronavirus disease 2019 (COVID-19) vaccination and to identify self-reported social behaviors that may affect the likelihood of recent or past SARS-CoV-2 infection. A cross-sectional, saliva-based serological study of 2,880 residents of Howard County, Maryland, was carried out from July through September 2021. Natural SARS-CoV-2 infection prevalence was estimated by inferring infections among individuals according to anti-nucleocapsid immunoglobin G levels and calculating averages weighted by sample proportions of various demographics. Antibody levels between BNT162b2 (Pfizer-BioNTech) and mRNA-1273 (Moderna) recipients were compared. Antibody decay rate was calculated by fitting exponential decay curves to cross-sectional indirect immunoassay data. Regression analysis was carried out to identify demographic factors, social behaviors, and attitudes that may be linked to an increased likelihood of natural infection. The estimated overall prevalence of natural infection in Howard County, Maryland, was 11.9% (95% confidence interval, 9.2% to 15.1%), compared with 7% reported COVID-19 cases. Antibody prevalence indicating natural infection was highest among Hispanic and non-Hispanic Black participants and lowest among non-Hispanic White and non-Hispanic Asian participants. Participants from census tracts with lower average household income also had higher natural infection rates. After accounting for multiple comparisons and correlations between participants, none of the behavior or attitude factors had significant effects on natural infection. At the same time, recipients of the mRNA-1273 vaccine had higher antibody levels than those of BNT162b2 vaccine recipients. Older study participants had overall lower antibody levels compared with younger study participants. The true prevalence of SARS-CoV-2 infection is higher than the number of reported COVID-19 cases in Howard County, Maryland. A disproportionate impact of infection-induced SARS-CoV-2 positivity was observed across different ethnic/racial subpopulations and incomes, and differences in antibody levels across different demographics were identified. Taken together, this information may inform public health policy to protect vulnerable populations.

**IMPORTANCE** We employed a highly innovative noninvasive multiplex oral fluid SARS-CoV-2 IgG assay to ascertain our seroprevalence estimates. This laboratory-developed test has been applied in NCI’s SeroNet consortium, possesses high sensitivity and specificity according to FDA Emergency Use Authorization guidelines, correlates strongly with SARS-CoV-2 neutralizing antibody responses, and is Clinical Laboratory Improvement Amendments-approved by the Johns Hopkins Hospital Department of Pathology. It represents a broadly scalable public health tool to improve understanding of recent and past SARS-CoV-2 exposure and infection without drawing any blood. To our knowledge, this is the first application of a high-performance salivary SARS-CoV-2 IgG assay to estimate population-level seroprevalence, including identifying COVID-19 disparities. We also are the first to report differences in SARS-CoV-2 IgG responses by COVID-19 vaccine manufacturers (BNT162b2 [Pfizer-BioNTech] and mRNA-1273 [Moderna]). Our findings demonstrate remarkable consistency with those of blood-based SARS-CoV-2 IgG assays in terms of differences in the magnitude of SARS-CoV-2 IgG responses between COVID-19 vaccines.

## INTRODUCTION

Because of frequent cases with mild or no symptoms, the number of reported coronavirus disease 2019 (COVID-19) cases does not reflect the true prevalence of severe acute respiratory syndrome coronavirus 2 (SARS-CoV-2) infections (1). In March 2020, the CDC conducted a study in 10 communities across the United States, documenting seropositivity rates 6 to 24 times greater than the rates of reported COVID-19 cases, indicating much higher levels of virus transmission than previously understood (1, 2). Understanding infection prevalence, particularly within community subpopulations, can inform public health policymakers as they seek to support high-risk groups with targeted public health programs (3, 4). A national American Red Cross serology study of blood donations from July 2020 through May 2021 estimated that the infection-induced seroprevalence rate of Hispanics was 30.0% (95% confidence interval [CI], 28.7% to 31.4%) and non-Hispanic Blacks (Blacks) was 21.1% (95% CI, 19.4% to 23.0%). These rates were higher than those of non-Hispanic Asians (Asians) 13.0% (95% CI, 11.7% to 14.3%) and of non-Hispanic Whites (Whites) 18.5% (95% CI, 18.2% to 18.8%) (5).

Throughout the course of the SARS-CoV-2 pandemic, the Howard County, Maryland (“Howard County”), Government and Howard County Health Department have worked together to minimize SARS-CoV-2 virus transmission and direct resources to vulnerable residents. An analysis of Howard County COVID-19 Dashboard case data from March 15 through November 30, 2020, indicated that, compared with White and Asian populations, the Hispanic and Black populations were overrepresented in the fraction of COVID-19 cases, given their representation in the general population (T. A. Colella, M. S. Robinette, H. P. Cowley, P. T. Koshute, A. C. Brown, S. V. Patel, S. R. Gravelyn, unpublished). A serology study was carried out from early July through mid-September 2021 to infer prior exposure to SARS-CoV-2, infection-induced (collectively referred to as “natural infection”) seropositivity, or vaccine-induced seropositivity in the general Howard County population and across different racial/ethnic subpopulations.

## RESULTS

The estimated overall prevalence of natural infection for Howard County was 11.9% (95% CI, 9.2% to 15.1%). This result suggests that there was a greater prevalence of natural infection within the community compared with reported case prevalence, which at the end of the sample collection period was 7%. This estimate accounts for discrepancies in sampled and actual proportions of age, race, and sex groups. However, the study population was not fully representative of the Howard County population geography. The majority of study participants came from central, urban zip codes rather than more rural parts of the county. Blacks and young adults were underrepresented, with study participants skewing older and White (see Fig. S1 in the supplemental material).

**FIG 1:**
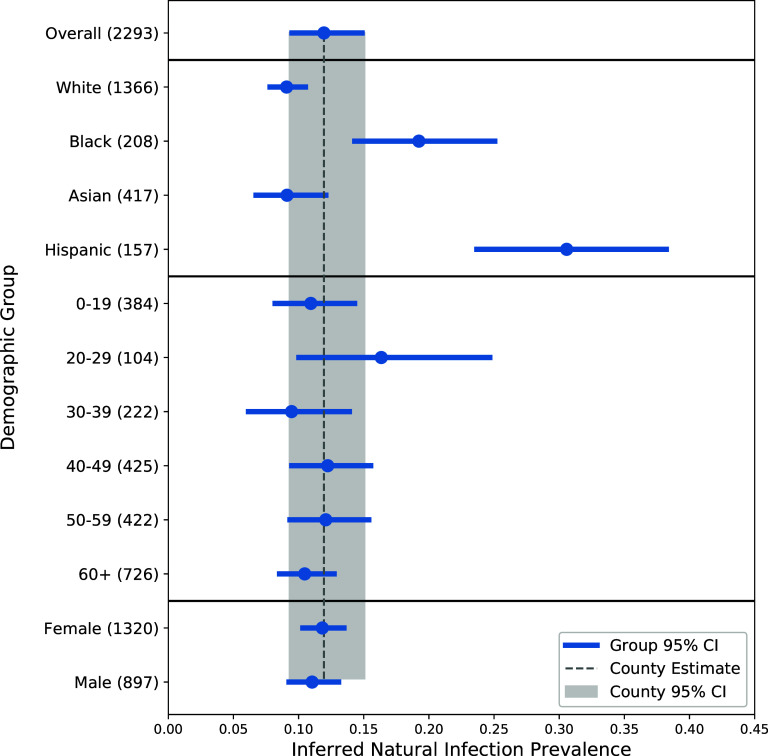
Inferred natural infection prevalence and 95% confidence intervals for race/ethnicity, age, and sex demographic groups. Intervals were compared with the overall estimated natural infection prevalence and 95% confidence interval, calculated from weighted sum of estimates of each demographic group. Numbers in parentheses are demographic group sample sizes.

As shown in Fig. 1, the prevalence of inferred natural infection was evaluated according to race/ethnicity, age, and sex. Race/ethnicity was correlated with inferred natural infection. Specifically, the infection-induced seroprevalence rate of Hispanics was 30.6% (95% CI, 23.5% to 38.4%), of Blacks was 19.2% (95% CI, 14.1% to 25.3%), of Whites was 9.1% (95% CI, 7.6% to 10.7%), and of Asians was 9.1% (95% CI, 6.5% to 12.3%). Compared with the overall county estimate, the prevalence of inferred natural infection was found to be slightly higher for participants in their 20s (16.3% [95% CI, 9.8% to 24.9%]), although no significant age effects were observed. To ensure that the trend among Hispanics was not driven by age distribution, inferred infections were analyzed across age groups for each race/ethnicity group. The infection prevalence among participants in their 20s was not limited to Hispanics; this age group had the highest infection prevalence for each race/ethnicity group except for Asians. Finally, sex was not found to be correlated with the risk of natural infection. For comparison, an alternative figure including 99.9% confidence intervals is included as Fig. S2.

Occupation, income, behaviors, and attitudes may affect the risk of becoming infected with SARS-CoV-2 (6–9). Based on household survey results, no occupation was found to be correlated with inferred natural infection. Similarly, study participants who reported having a public-facing job across all occupations were not found to have an increased prevalence of an inferred natural infection. Finally, informed by sample collection location, samples collected from Howard County residents who worked at Howard County police and fire stations (*n* = 131) determined that these first responders had a natural infection prevalence of 4.5%, which is lower than the overall study population’s natural infection prevalence.

Study participants were not surveyed regarding household income. Instead, participants’ household addresses were used to identify corresponding census tracts and average household income. Census tracts were chosen as a representative aggregate of participant addresses because they represent generally homogeneous socioeconomic backgrounds while still allowing differences to be identified across census tracts. A significant difference was found for census tract income between households with and without one or more inferred natural infections among household participants (Mann-Whitney *U* test; *P* = 0.0006), with households from census tracts with lower average income having a higher prevalence of natural infection.

**FIG 2:**
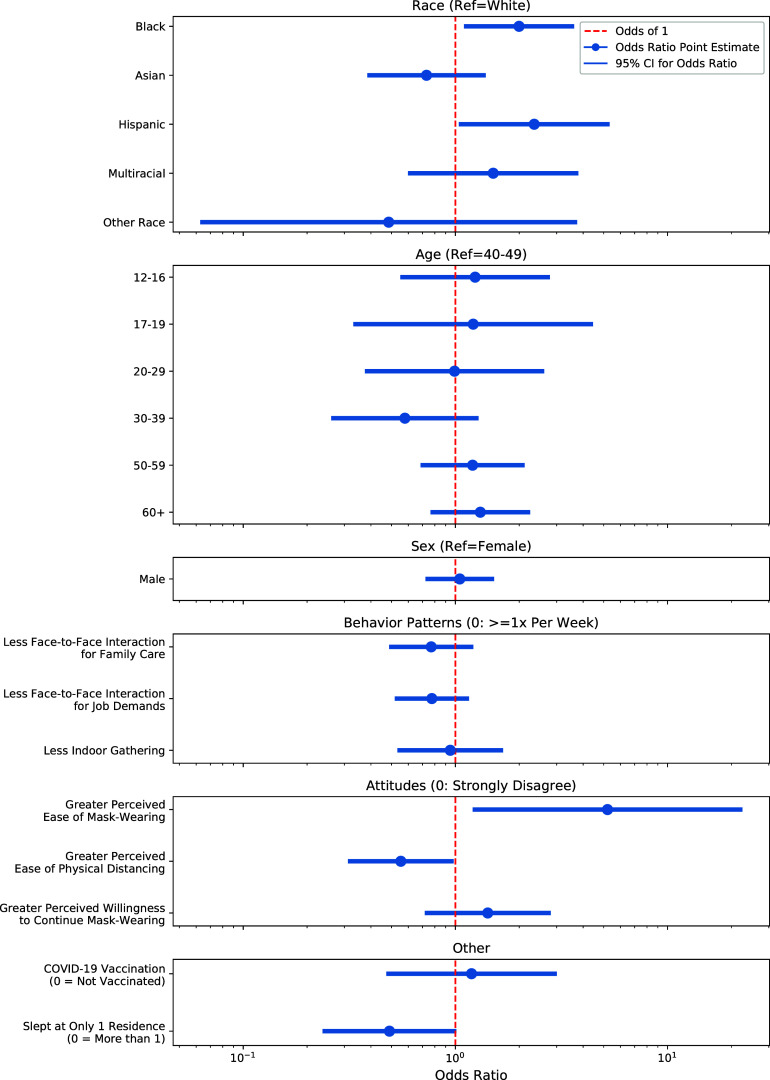
Effect of surveyed behaviors and beliefs on odds of inferred natural infection (displayed on a log-scale *y* axis). The model coefficients and 95% confidence intervals were exponentiated to create odds ratios for natural infection relative to the reference category listed on the *y* axis.

A logistic regression model was constructed to investigate the potential effect(s) of behaviors and attitudes on the likelihood of contracting a COVID-19 infection, while controlling for the effects of demographic variables and vaccination status. After correcting for multiple comparisons (12 total) using a Bonferroni correction, the significance threshold was set to *P* = 0.004. No statistically significant effects were identified at this threshold. Figure 2 shows the odds ratios for each covariate with a 95% confidence interval. The same odds ratios with a 99.9% confidence interval can be found in Fig. S3. The odds ratio for the perceived ease of mask wearing has a very large confidence interval due in part to the uneven scaling from exponentiating larger values. The odds ratio for perceived ease of mask wearing is also larger than the odds ratio for willingness to continue wearing masks, even when they are not explicitly required. While perceived ease of mask wearing and perceived ease of physical distancing were significant factors at the *P* = 0.05 level, they were not significant after correcting for multiple comparisons. Similarly, the coefficients of a generalized linear mixed model (GLMM) model were not significant after correcting for multiple comparisons (see Fig. S4 and S5).

**FIG 3:**
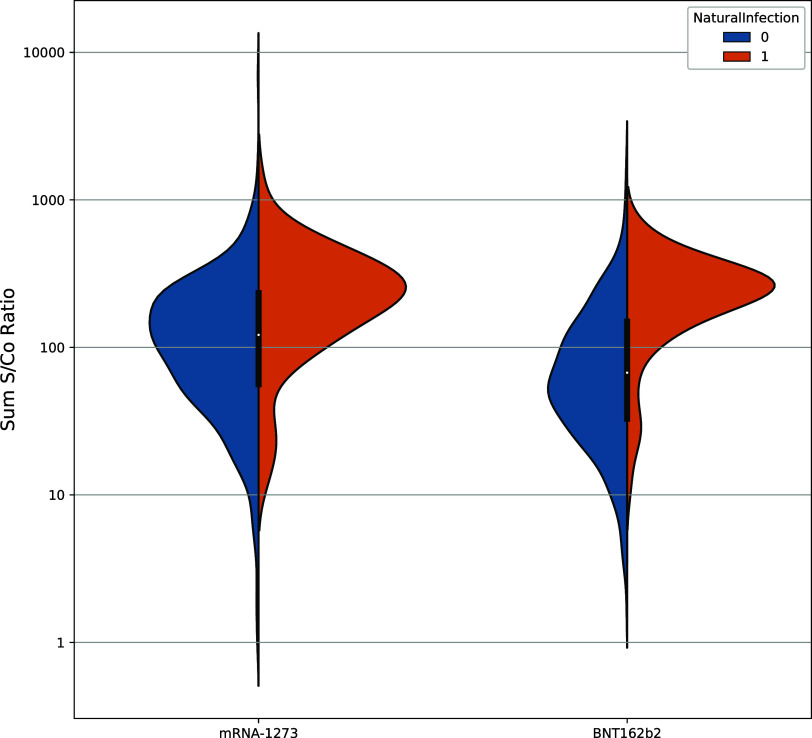
Antibody levels in mRNA-1273 and BNT162b2 vaccine recipients and individuals who were negative (0) (mRNA-1273, *n* = 451; BNT162b2, *n* = 617) and positive (1) (mRNA-1273, *n* = 60; BNT162b2, *n* = 81) for an inferred SARS-CoV-2 infection.

At the end of the sample collection period, the SARS-CoV-2 vaccination rate of the general population was 71% compared with 86% of the study population. This overrepresentation of vaccinated participants relative to the general population was observed across all demographics. The majority of vaccinated study participants reported having received either the mRNA-1273 or the BNT162b2 vaccine. As shown in Fig. 3, participants who reported having received the mRNA-1273 vaccine had 50.0% higher antibody levels compared with BNT162b2 vaccine recipients at the time of collection (mRNA-1273 sum of signal to cutoff [S/Co] ratio geometric mean: 90.4, BNT162b2 S/Co ratio geometric mean 60.4). This figure includes data from participants whose samples were collected between 74 and 194 days after the first day of the month of their reported first dose. A comparable figure excluding participants with longer intervals between first doses and sample collection is included in Fig. S7. Notably, of the study participants who reported vaccination, 6.5% tested negative for SARS-CoV-2 antibodies. Seronegative individuals may produce antibodies that do not pass the seropositivity threshold. Although some of these individuals may have been immunocompromised, 75% (*n* = 72) were vaccinated between 4 to 7 months before study participation, as seen elsewhere (10).

Antibody level regression model results showed that the exponential decay term was significant (*P* < 0.001). However, the interaction between time since first vaccination dose and vaccine type was not significant (*P* = 0.71); therefore, the regression does not show a significant difference between decay rates for different vaccine types. Regression results indicate a half-life for the sum of normalized anti-receptor-binding domain (anti-RBD) and anti-spike (anti-S) antibodies of about 140 days (95% CI, 100 to 240 days). The antibody totals were normalized by cutoffs determined in a prior study (11). This result is similar to a longitudinal saliva study (12), which found an estimated half-life of 100 days for anti-RBD immunoglobulin G (IgG) (95% CI, 58 to 141 days) and 148 days (95% CI, 62 to 238 days) for anti-S IgG responses in saliva, consistent with half-life estimates previously reported in blood (13).

Consistent with Fig. 3, the regression showed a significant difference in antibody response between mRNA-1273 and BNT162b2 (*P* < 0.001) recipients. Thus, the antibody levels following mRNA-1273 vaccination remain at higher levels for at least 195 days. Coefficients from models with and without the interaction term (time since first dose × vaccine type) are included in Table S4 and S5.

Inferred natural infection and age had a significant effect on antibody levels (*P* < 0.001 for both). Participants who received the mRNA-1273 or the BNT162b2 vaccine and were classified as having an inferred natural infection had higher antibody levels than those who were vaccinated but did not have an inferred natural infection. Additionally, younger participants had higher antibody levels than older participants. Participant sex was found to have a minor effect on antibody levels (*P* = 0.03), with females having higher antibody levels. Participant race/ethnicity had no effect on antibody levels, with *P* = 0.36, 0.53, and 0.94 for Black, Hispanic, and Asian participants, respectively, using White race/ethnicity as a reference.

## DISCUSSION

The estimated overall prevalence of natural infection for Howard County, Maryland, was 11.9% (95% CI, 9.2% to 15.1%), compared with 7% reported COVID-19 cases. Antibody prevalence indicating natural infection was highest among Hispanic and Black participants and lowest among White and Asian participants. Additional risk factors for natural infection included lower census tract household income. Recipients of the mRNA-1273 vaccine had higher antibody levels than those of BNT162b2 vaccine recipients. Older study participants had overall lower antibody levels compared with younger study participants.

The Howard County serology study used the saliva-based JH-EHMIL test for SARS-CoV-2 antibody detection. Although a limitation of (all) antibody binding assays is that it is not known if the detected IgG can neutralize SARS-CoV-2 nor whether it is sufficient to confer protection against reinfection, this test offers a noninvasive alternative to traditional, blood-based antibody testing that is easily deployable in a community setting, thereby offering insights into population seropositivity.

There were several additional important limitations of this study. Of the 2,434 samples with sufficient IgG concentration, only 2,293 participants had a complete data set that included serology data linked to deidentified individual participant surveys. Of these, only 1,441 participants were linked to household survey data, allowing for analysis of seroprevalence and natural infection status in combination with both demographic information reported behaviors. Data collected via surveys, namely, month of first vaccine and month of confirmed infection, were self-reported by participants, largely based on memory and lacked specific dates.

Despite the large number of study participants, the study population is a self-selected nonrandomized sample and thus is not fully representative of the Howard County community. Young adults are significantly underrepresented (*P* < 0.001); this shortage may have been remedied by sampling at the community college beyond the summer months. Black study participants were also significantly underrepresented by 55% (*P* < 0.001). Most Black study participants were sampled during targeted sample collection events held at faith-based communities. These communities returned to in-person worship services toward the end of the sample collection period, and most of these services were limited, ticketed events. The general public sample collection events attracted older White participants, who are overrepresented in the study population (*P* < 0.001). These participants expressed a strong desire to understand their level of protection against COVID-19, motivated by concerns regarding waning antibodies or underlying health conditions. Vaccinated residents are also overrepresented in the study population. Data analysis techniques for adjusting for nonrepresentative samples were used to account for these discrepancies.

Another limitation of this study was antibody detection relative to the timing of natural infection due to antibody decay. Of the study participants who self-reported a lab-confirmed infection (*n* = 123), only 72% (*n* = 89) were still found to have antibodies to the nucleocapsid (N) protein. Of those individuals for whom antibodies were not detected (*n* = 34), all except two reported infection more than 6 months before sample collection.

Additional analysis of behavioral responses found that greater perceived ease of mask wearing was positively associated with natural infection. This result may be due to individuals who are less likely to go out into the community because they find masks uncomfortable and thus have a decreased risk of infection, whereas those who find mask wearing easy may be wearing masks improperly. Analysis of behavioral responses also found that greater perceived ease of physical distancing was negatively associated with natural infection. Taken together, these results suggest that the perceived ease of these two protective behaviors may not provide the same results with respect to acquiring a natural infection. Follow-on work to tease out these potential relationships could explore this hypothesis. The understanding of such behaviors among the population will continue to be important as new, highly transmissible SARS-CoV-2 variants continue to spread throughout the region and the country.

Despite the wealth of Howard County, which has a low social vulnerability index (overall score 0.1242, on a scale of 0 [lowest] to 1 [highest]), the pandemic has disproportionately impacted Black and Hispanic Howard County residents, as evidenced by both reported COVID-19 cases and inferred natural infections. Quantifying this disparity in the community is a critical step toward informing public health policy and equitably directing resources to populations that have the greatest need. Additionally, identifying vulnerable populations that lack antibody protection can help inform targeted public health communications.

## MATERIALS AND METHODS

### Saliva sample collection.

Saliva samples were self-collected by Howard County residents at 36 events held from July 4 through September 19, 2021, at different times of day, days of the week, and locations throughout the county, including libraries, faith centers, parks, community centers, and government buildings. Participation was voluntary, and the study protocol was approved by the Johns Hopkins University Institutional Review Board. There was no age limitation for study participation, and test results were mailed to study participants within 8 weeks of sample collection. Information about the study was disseminated via the Howard County Government website and social media channels. Faith community leaders and members of Howard County Government workgroups representing racial and ethnic subpopulations encouraged community participation. Study facilitators coached participants in saliva sample self-collection. Reflecting the diversity of Howard County, Spanish, Korean, and Mandarin translators were available as needed. Because each region has distinct demographics, these results are not intended to be generalized beyond Howard County.

### Summary of data filtering.

**FIG 4:**
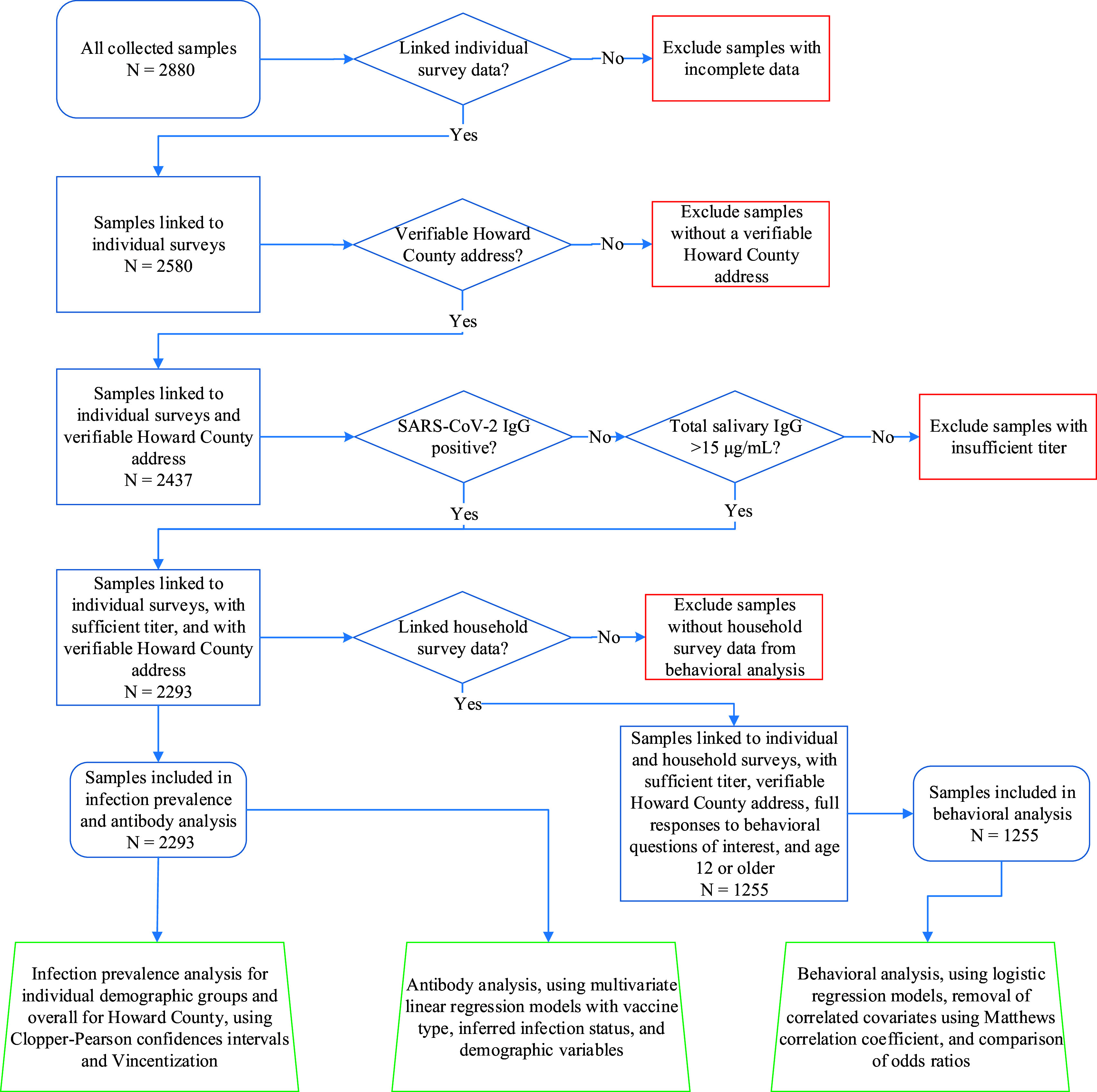
Sample-filtering process for inclusion in data analyses.

Total collected saliva samples (*n* = 2,880) were restricted to 2293 samples used for analyses following the data filtering process illustrated in Fig. 4. First, saliva samples that could not be linked to individual study participant surveys, described below, were excluded. Of the remaining samples, those without a verifiable Howard County address were removed. Finally, among 2437 samples from participants with verifiable Howard County addresses, 144 (5.9%) were excluded because they had an insufficient IgG concentration (described below as “indeterminate” samples).

### Saliva sample processing and multiplex SARS-CoV-2 IgG assay.

Samples were processed and analyzed as previously reported in a Clinical Laboratory Improvement Amendments of 1988 (CLIA) laboratory setting (14–17). Briefly, saliva samples were tested using a laboratory-developed test under CLIA/College of American Pathologists guidelines. This assay was based on a protocol developed by the Johns Hopkins Environmental Health Microbiology and Immunology Laboratory (JH-EHMIL) (11). The testing included 7 SARS-CoV-2 antigens (2 nucleocapsid [N], 3 receptor-binding domain [RBD], and 2 spike [S] antigens) to measure IgG binding to SARS-CoV-2.

A recent study found that this assay has 98.8% sensitivity and 100% specificity. Moreover, this assay is comparable to a serum-based assay with Spearman correlation values between the two assays’ IgG responses to various antigens generally above 0.75 (11). Another study found that the positive percent agreement between the saliva- and serum-based assays consistently exceeded 90% (16).

Antibody measurements were normalized using summary metrics from samples collected during prepandemic cohort studies and compared with established thresholds (11, 14). Samples with SARS-CoV-2 IgG signal (anti-N, anti-RBD, and anti-S IgG combined) above the threshold were classified as positive. Samples with IgG signals below the threshold with insufficient total IgG were considered indeterminate and excluded from analyses (to improve sensitivity). Individuals who had prior exposure or were recently naturally infected (collectively referred to as “inferred natural infection”) are seropositive for both SARS-CoV-2 spike S/RBD and N proteins. In contrast, the mRNA-1273 and BNT162b2 vaccines are based on the SARS-CoV-2 genetic instructions for making the S/RBD protein but not the N protein. Thus, individuals who received a SARS-CoV-2 vaccine, without prior exposure or recent natural infection, have antibodies specific for the S/RBD protein but not the N SARS-CoV-2 protein.

These considerations led to the following sample classification logic:

- Anti-SARS-CoV-2 (N/RBD/S) IgG negative but insufficient total IgG → INDETERMINATE;
- Anti-SARS-CoV-2 (N/RBD/S) IgG negative with sufficient total IgG → NEGATIVE ANTIBODY RESULTS and NO INFERRED NATURAL INFECTION;
- Anti-SARS-CoV-2 (N/RBD/S) IgG positive AND anti-N IgG negative → POSITIVE ANTIBODY RESULTS (i.e., indicates vaccination) and NO INFERRED NATURAL INFECTION; and
- Anti-SARS-CoV-2 (N/RBD/S) IgG positive AND anti-N IgG positive → INFERRED NATURAL INFECTION with or without vaccination.

### Estimating prevalence of prior SARS-CoV-2 infection.

By analyzing trends in the proportions of participants with each of the four outcomes described above for various demographic groups, immunological variation was characterized across the county. The supplemental material provides an analysis of how well study participants represent the county according to age, race/ethnicity, and sex. To estimate the overall infection prevalence, the prevalence of each demographic group was weighted according to the proportion of each demographic group in the population of Howard County as reported in the 2019 American Community Survey (18).

For each demographic group, it was assumed that participants represent a random sample of the corresponding population. The confidence intervals for groups were calculated using the Clopper-Pearson method (19). For each demographic variable (age, race, and sex), a weighted average of each group’s infection prevalence was computed according to each group’s population proportion (18). Corresponding confidence intervals were computed using Vincentization (20), weighting by county proportion for each group. To obtain a single overall county estimate and confidence interval, unweighted averages of each of the 3 separate point estimates and interval bounds were computed.

To show relative differences between groups, 95% confidence intervals are shown. However, as correction for multiple comparisons, significant effects are identified only at the 99.9% confidence level (1 to 0.05/30 ≈ 0.999 for approximately 30 comparisons through the study).

### Antibody decay rate estimation.

To estimate SARS-CoV-2 antibody decay rates following vaccination, it was assumed that each participant experienced an exponential decay in antibody levels over time. Multivariate regression models were fitted to log_10_-transformed normalized anti-RBD + anti-S IgG levels using the following covariates: estimated time since first vaccination dose; binned age (20 to 39, 40 to 59, and 60+); sex; vaccine type (BNT162b2 and mRNA-1273); race; inferred natural infection status; and estimated time since first dose × vaccine type interaction. The latter regression model’s interaction term was used to test whether the two vaccine types yield significantly different antibody decay rates.

Participants reported the month in which they received their first vaccination dose. To focus on participants that were fully vaccinated, this analysis excluded samples that were collected fewer than 74 days since the first day of the reported month of the first dose. To ensure strong *Anti-SARS-CoV-2 (RBD/S) IgG* levels, samples that were collected more than 194 days since the first day of the reported month of the first dose were also excluded. This epoch was used for the calculation of mean antibody levels, and antibody decay rates, for those participants who reported having received either the mRNA-1273 or the BNT162b2 vaccine.

### Survey analysis: behaviors and attitudes.

Individual participant surveys were completed at the time of sample collection. These surveys collected demographic information and vaccination status. One member of each household completed an additional survey addressing household attitudes toward SARS-CoV-2 testing and vaccination, as well as personal and social behaviors before and after SARS-CoV-2 vaccines were made widely available. Surveys were provided in English, Spanish, Korean, and Mandarin in paper format as well as through an online Qualtrics form. Serology data, household survey data, and individual participant survey data were matched together, yielding 1,441 participants for which all three types of data were complete. After removing samples from participants younger than 12 years old (for whom SARS-Cov-2 vaccines were not available at the time of the study), there were 1,255 samples paired with household surveys available for further study (see Fig. 4).

There is substantial evidence that protective behaviors correlate with decreased risk of infection, including social distancing (21), proper hand washing (22), and mask wearing (23). Therefore, protective behaviors and attitudes were evaluated as potential predictors of inferred natural infection. Participants’ answers were transformed to a numeric variable, with 1 being presumed more protective against SARS-CoV-2 infection and 0 being less protective (see Table S2). From this scoring, generalized linear models were created to study the association between demographics, behaviors, and attitudes and natural infection status in Howard County. The demographic covariates included in the model were age, race/ethnicity, and sex. Because of a small sample size (*n* = 2), Native American/Native Alaskan participants were excluded from the modeling analysis. Individual vaccination status was included as a covariate to control for its effect.

To control for multicollinearity, Matthew’s correlation coefficient was used to identify instances of high correlation (ɸ > 0.30). This resulted in the removal of frequency of indoor dining (correlated with indoor gathering) and ease of access to COVID-19 vaccination (correlated with greater perceived ease of mask wearing). There was a high correlation between participants under the age of 12 and vaccination status, reflecting that at the time of study children under 12 were not eligible for COVID-19 vaccination. Given this high correlation, children in this age group were excluded from the modeling analysis. Models for classifying binary natural infection status (1 = naturally infected) were fit using the Python library *statsmodels* (24). The resulting coefficients for each covariate were exponentiated to yield the odds ratio for natural infection for each covariate relative to the reference category.

Survey responses and natural infection status exhibit a dependence structure within these data set because multiple individuals from the same household often provided samples. Moreover, participants sampled at particular community sites may also have correlated outcomes. The potential effect of this dependence within household and within collection site was explored using GLMM with collection site category and household modeled as nested random effects. Participants sampled on the same date or at the same site were grouped into a common collection site category. Samples’ collection sites were not directly tracked. However, since samples were collected only at a single site on all but two dates, this site category roughly corresponds to individual sites. In both models, significant effects were identified at the 99.9% level to correct for multiple comparisons.
