# Supplementary material for: A Saliva-Based Serological and Behavioral Analysis of SARS-CoV-2 Antibody Prevalence in Howard County, Maryland
Source: Microbiol Spectr. 2023 Jun 8;11(4):e02765-22. doi: 10.1128/spectrum.02765-22 (PMC10433989; doi:10.1128/spectrum.02765-22)
Supplement: Supplemental file 1 — Supplemental text, Tables S1 to S5, and Fig. S1 to S7. Download spectrum.02765-22-s0001.pdf, PDF file, 1.5 MB [file spectrum.02765-22-s0001.pdf]

SUPPLEMENTAL MATERIAL

METHODS

To meet the primary objective of the study, a significant effort was made to collect samples from different racial and ethnic groups. Given that minority populations generally live in the central, more densely populated parts of the county, individuals residing in these zip codes were over-sampled relative to the general population, whereas individuals residing in the more rural, western part of the county were under-sampled.

The *County Population* in Table S1 is the number of Howard County residents in each ZIP Code.[1,2] The *Samples Collected* column lists the number of samples processed that could be definitively associated with each ZIP code based on collected demographic data. The *Representative?* column indicates whether the number of samples is representative of the Howard County population in that ZIP code at the 95% confidence level based on a binomial confidence interval (CI) given the Howard County population in that ZIP Code. The *Difference* column is the delta between the number of samples collected and the expected number of samples given the county population, which is expressed as a percentage of the expected number of samples in the *% Difference* column.

Table S1 — Geographical Representation of Study Participants Relative to Howard County, Maryland, General Population.

ZIP	County Population	Samples Collected	Representative?	Difference	% Difference
20759	5311	78	over	48	156%
21046	15655	161	over	71	79%
21029	10895	111	over	48	77%
21042	41195	397	over	160	68%
21045	40365	370	over	138	60%
21044	44256	400	over	146	57%
21043	47349	332	over	60	22%
20777	3687	23	expected	2	9%
21163	7030	42	expected	2	4%
20763	1635	9	expected	0	−4%
20723	35411	194	expected	−9	−5%
21737	2462	10	expected	−4	−29%
21036	2112	8	expected	−4	−34%
21075	33726	165	under	−29	−15%
21104	5762	24	under	−9	−27%
21738	3827	13	under	−9	−41%
21797	8873	24	under	−27	−53%
21794	2277	6	under	−7	−54%
20794	17939	30	under	−73	−71%
21723	759	1	under	−3	−77%
21076	17442	15	under	−85	−85%
21771	29901	10	under	−162	−94%
21784	37365	4	under	−211	−98%
20833	7369	0	under	−42	−100%
20701	92	0	under	−1	−100%

The age representation of the study population relative to the general Howard County population was evaluated.[3] Binomial tests confirmed the statistical significance of this imbalance for each group ($p<0.001$). As shown in Figure S1-A, the study population over-sampled residents from

older age groups and under-sampled residents from younger age groups. To address the under-representation of young residents, saliva sample collection events were held at locations where children would be present, for example, during dismissal of summer camps at a local community center and before and after children's library classes. The over-representation of older residents was due to expressed concerns of waning antibody levels and feelings of vulnerability because of co-morbidities. The demographic imbalance of study participants may have affected the precision of the antibody decay analysis. As shown in Figure S2-B, the study population over-sampled female residents and under-sampled male residents. This skewing towards female participation in voluntary studies has been observed elsewhere.[4]

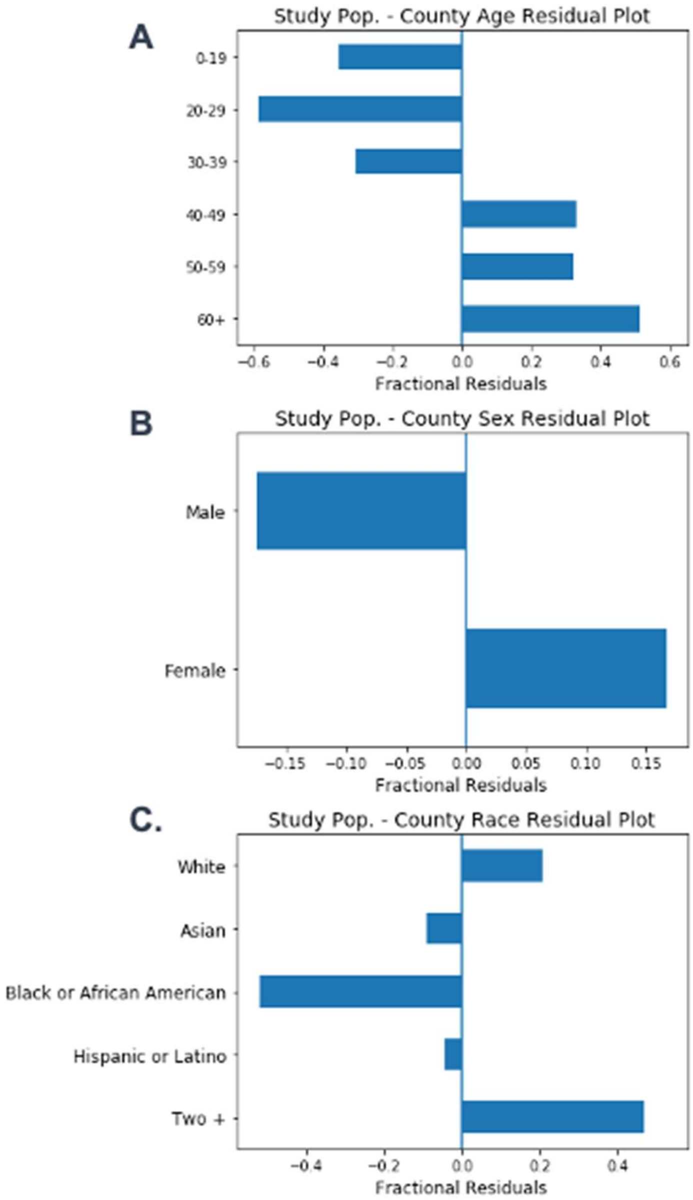

Figure S1 — Age (A), Sex (B), and Racial and Ethnic (C) Representation of Study Population Relative to General Population of Howard County, Maryland

About 50% of Howard County residents are White, 19% Black, 18% Asian, and 7% Hispanic.[1,2] All racial and ethnic groups are well represented in the study population relative to the Howard County general population, with the exception of the Black subpopulation, which is significantly under sampled by 55% (see Figure S1-C). Blacks were willing to participate in the study when sample collection events were hosted at their faith communities. However, during the majority of

the sample collection period, faith communities comprising primarily Black/African American congregants were holding live-streamed, virtual services. At the end of the sample collection period, sample collection events were held at in-person “welcome back” social events and services hosted by these faith communities. Many of these services were limited, ticketed events in order to maintain social distancing between attendees.

Table S2 — Individual and Household Risk Variables Scoring Rubric

<table>
<tr><th>Survey</th><th>Variable</th><th>Response Values</th><th>Score</th></tr>
<tr><td rowspan="4">Individual</td><td rowspan="2">Belief that individual would have a poor COVID-19 outcome</td><td>Yes</td><td>1</td></tr>
<tr><td>No or Unsure</td><td>0</td></tr>
<tr><td rowspan="2">Average number of residences slept per week</td><td>1 residence</td><td>1</td></tr>
<tr><td>More than 1 residence</td><td>0</td></tr>
<tr><td rowspan="4">Household</td><td rowspan="2">Frequency of behaviors:
– Indoor dining
– Indoor gathering
– Face to face interaction with others outside the home due to job demands
– Face to face interaction with others outside the home due to family care demands</td><td>Less than weekly</td><td>1</td></tr>
<tr><td>Weekly or more</td><td>0</td></tr>
<tr><td rowspan="2">Attitudes:
– Easy to abide by mask guidelines
– Easy to abide by physical distancing guidelines
– Easy to access COVID-19 vaccination
– Continue wearing masks even when not required</td><td>Agree, Strongly Agree, or Neutral</td><td>1</td></tr>
<tr><td>Disagree or Strongly Disagree</td><td>0</td></tr>
</table>

Answers to the following questions on the individual and household survey were used to create a score for how protective an individual’s behaviors and attitudes were during the COVID-19 pandemic. Responses were scored as below, with a value of 1 indicating that individuals responding in this way are either engaging in more protective behaviors or are hypothesized to engage in more protective behaviors.

RESULTS

The following table compares estimated infection prevalence by race/ethnicity group with data reported on the Howard County dashboard.[5] We inferred natural infections from antibody levels using the following criteria: *Anti-SARS-CoV-2 (N/RBD/S) IgG* > 10 AND *anti-N IgG* > 1.9. The reported COVID-19 case rates were calculated by dividing the number of cases reported for a given race/ethnicity group on the dashboard by the population estimate for each race/ethnicity group from the 2019 American Community Survey.[6] Because approximately 25% of all cases reported on the dashboard are listed as "Unknown" race/ethnicity, these calculated case rates represent lower bounds for each race/ethnicity group.

Table S3 — Inferred Natural Infection and Vaccination Proportions of Study Population, According to Race/Ethnicity

Race/Ethnicity Group	# Study Participants	Estimated Natural Infection Prevalence of Study Population [95% CI]	Reported COVID-19 Case Rate for General Population	Self-Reported Vaccination Proportion for Study Population
White	1366	0.091 [0.076, 0.107]	0.04	0.88
Asian	417	0.091 [0.065, 0.123]	0.03	0.84
Black/African American	208	0.192 [0.141, 0.253]	0.07	0.86
Hispanic/ Latino	157	0.31 [0.235, 0.384]	0.09	0.83
Other	145	0.083 [0.043, 0.140]	0.03	0.67
**Overall**	**2293**	**0.119 [0.092, 0.151]**	**0.07**	**0.86**

To account for multiple hypothesis tests throughout this paper, the Figure S2 shows inferred natural infection prevalence and 99.9% CI for each demographic group, as well as an overall county estimate and CI. With this stricter threshold for significance, we still see a higher prevalence in Hispanic study participants. All other relationships are within error bars of the county estimate.

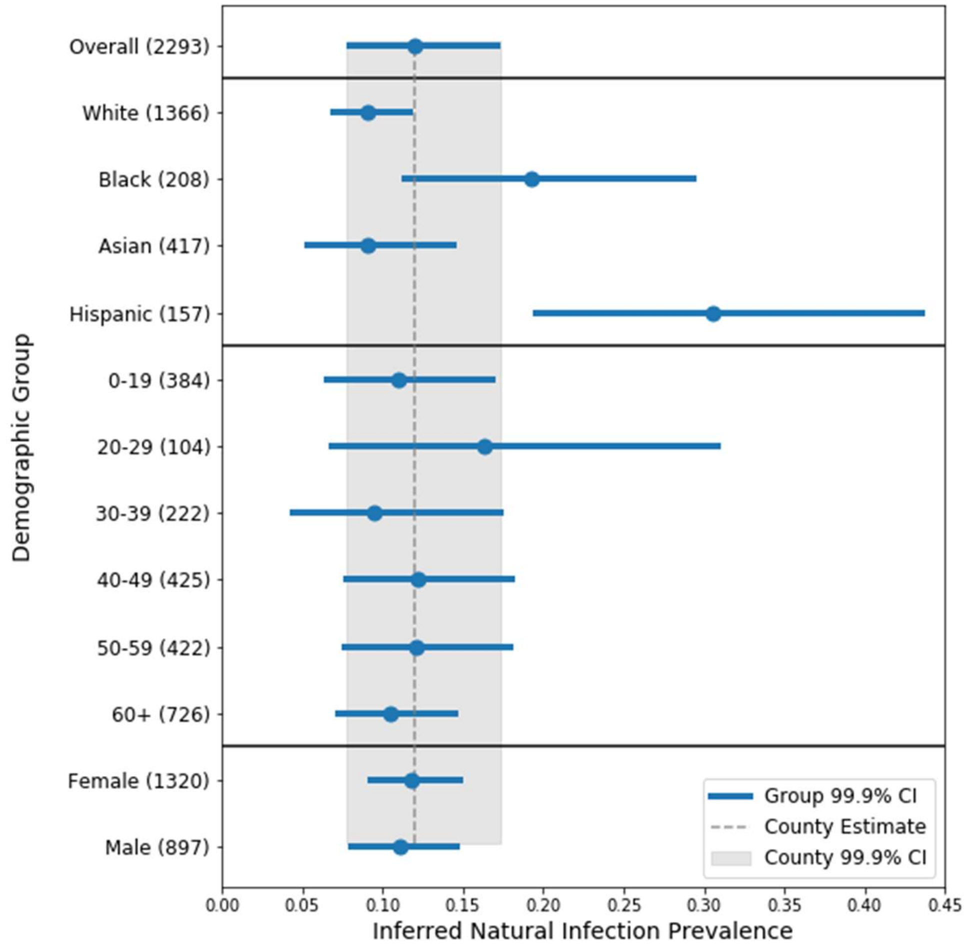

Figure S2 — Inferred Natural Infection Prevalence and 99.9% Confidence Intervals for Race/Ethnicity, Age, and Sex Demographic Groups (Intervals are compared with the overall estimated natural infection prevalence and 99.9% confidence interval, calculated from weighted sums of estimates of each demographic group. Numbers in parentheses are demographic group sample sizes.)

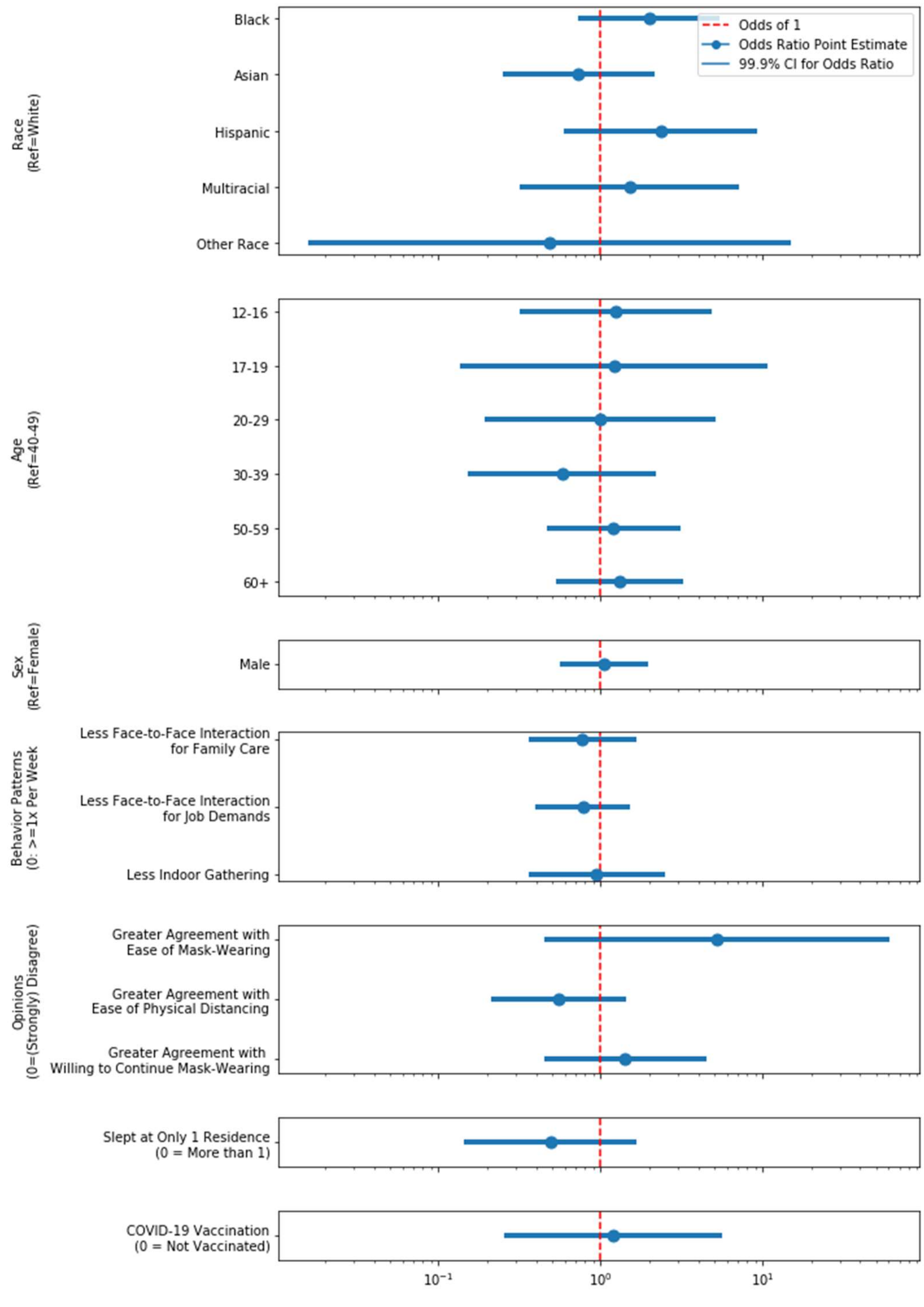

Figure S3 — Odds Ratios and 99.9% confidence intervals for Logistic Regression Model

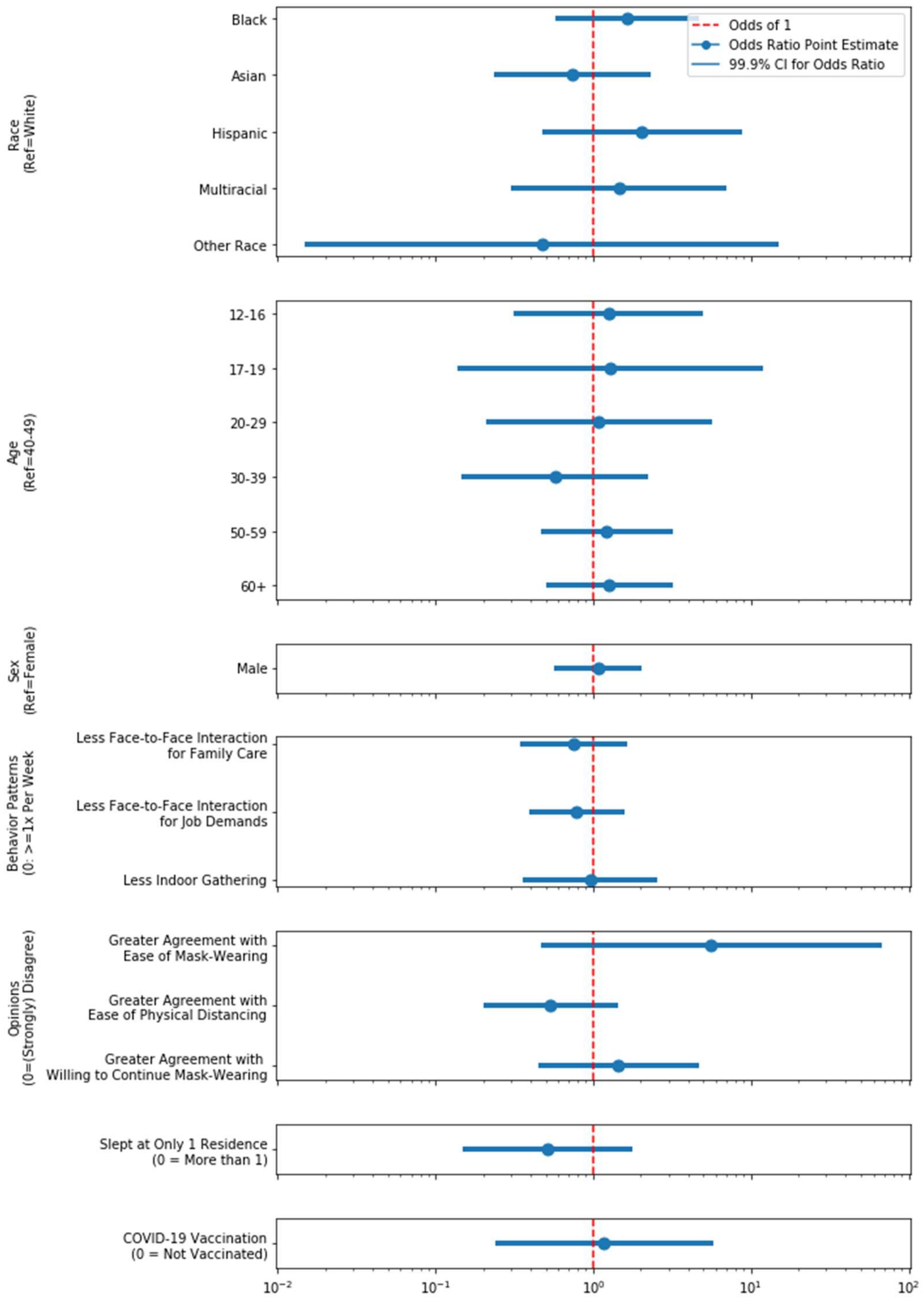

Figure S4 — Odds Ratios and 99.9% Confidence Intervals for Fixed Effects in GLMM with Collection Site Category and Household as Random Effects

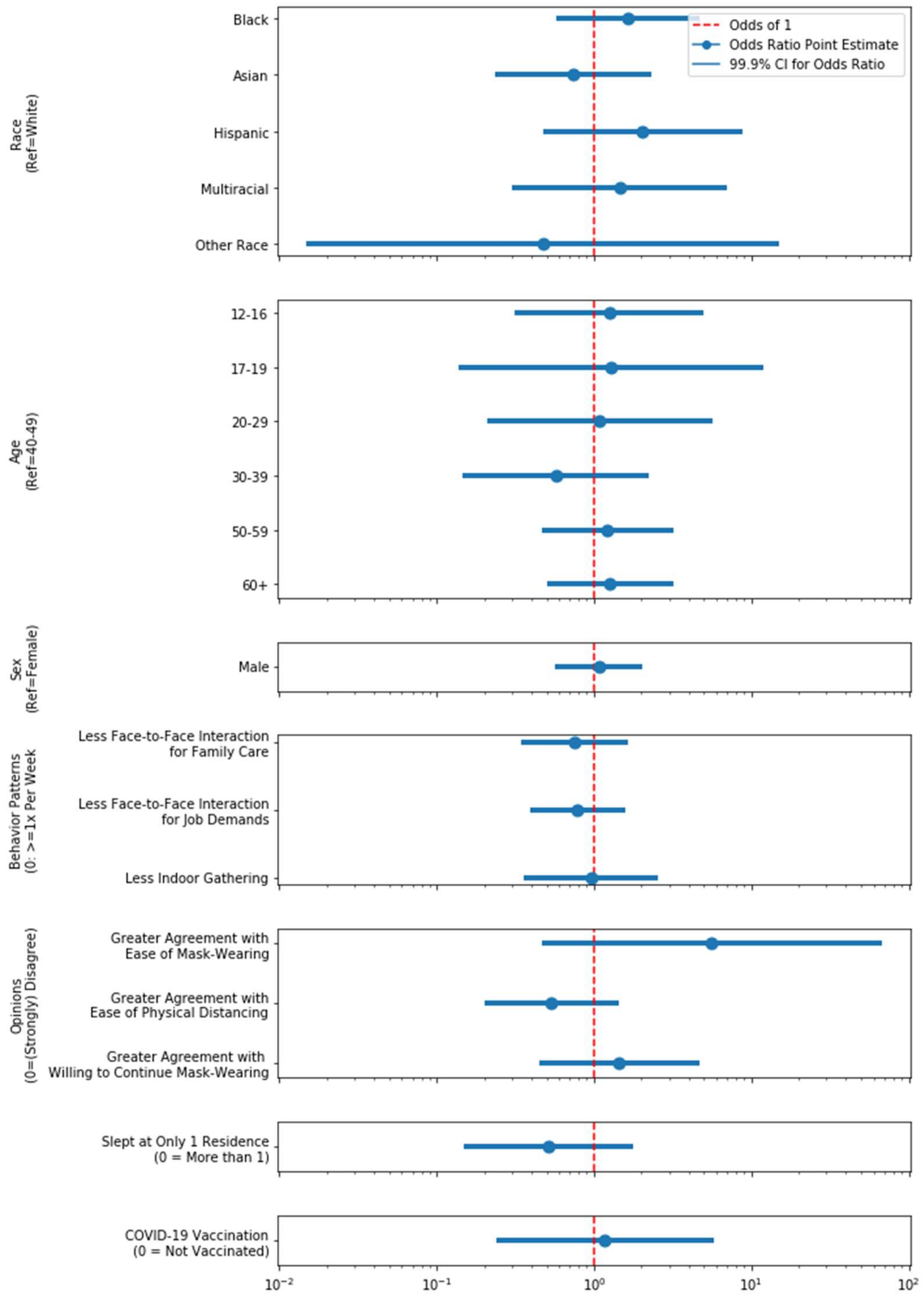

Figure S5 — Odds Ratios and 99.9% Confidence Intervals for Fixed Effects in GLMM with Collection Site Category as the Only Random Effect.

The following figure shows the antibody time-series data discussed in the main body, and gives additional information about the exponential fits to that data.

Figure S6 plots the aggregated antibody measure *anti-RBD* + *anti-S* versus time since first vaccination. The data in this figure is a subset of the study data, here we have only included participants vaccinated with mRNA-1273 or BNT162b2, and restricted the time epoch to be greater than 60, and less than 180, days since first vaccination. The main body of the paper discusses the logic for choosing this epoch, but in brief, this restriction aims to include only antibody measurements that will occur during the decay of participant antibody levels. In this analysis, days since first vaccination for a given participant is estimated as the 15th day of the month that the participant reported as the month of their first vaccination.

In Figure S6 the solid orange line shows an exponential fit to the data, with the fit coefficients shown in the upper right. Regression analysis discussed in the main body established that the decay rates, and corresponding half-lives, for mRNA-1273 or BNT162b2 were not distinguishable at the 95% confidence level. Because of this, the exponential decay model was fit to the combined mRNA-1273 or BNT162b2 data. The fit produced an exponential decay constant of -0.0076 $[days^{-1}]$, shown by the solid orange line in the figure. This decay constant, and associated standard errors, corresponds to a half-life of 91 days with 95% lower and upper CIs of 71 and 127 days, respectively. The upper and lower limits of the 95% CI for the fit are shown by orange dashed lines.

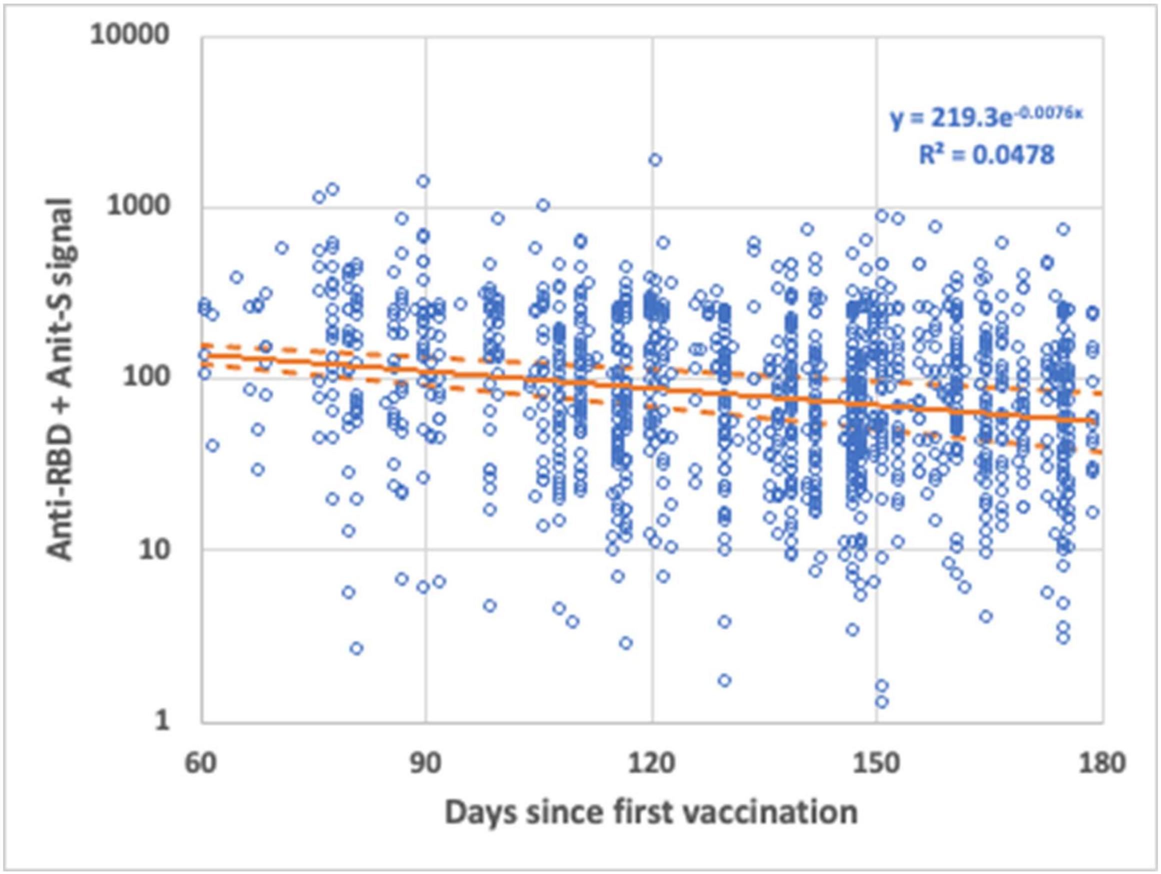

Figure S6 — Aggregated Antibody Measure anti-RBD + anti-S Decay for Participants Vaccinated with mRNA-1273 or BNT162b2

Table S4 — Linear Regression Coefficients for log10(RBD Signal/Cutoff Ratios + S Signal/Cutoff Ratios) Response Variable (including an interaction term between time since vaccination and vaccine type)

	**Coefficient**	**95% Confidence Interval**	**p-value**
Estimated time since vaccination (days)	−0.0024	−0.0037, −0.0011	< 0.001
Age	−0.09	−0.129, −0.055	< 0.001
Vaccine type			
BNT162b2	[Reference]		
mRNA-1273	0.197	−0.062, 0.456	0.14
Inferred natural infection	0.400	0.314, 0.484	< 0.001
Race			
White	[Reference]		
Black/African American	−0.045	−0.144, 0.053	0.37
Asian	0.002	−0.065, 0.068	0.95
Hispanic/Latino	0.032	−0.073, 0.139	0.55
Other race(s)	0.105	−0.014, 0.224	0.08
Sex			
Male	[Reference]		
Female	0.059	0.007, 0.112	0.03
Estimated time since vaccination * vaccine type	0.0004	−0.0015, 0.0022	0.71

Table S5 — Linear Regression Coefficients for log10(RBD Signal/Cutoff Ratios + S Signal/Cutoff Ratios) Response Variable (not including an interaction term between time since vaccination and vaccine type)

	**Coefficient**	**95% Confidence Interval**	**p-value**
Estimated time since vaccination (days)	−0.002	−0.003, −0.001	< 0.001
Age	−0.092	−0.129, −0.056	< 0.001
Vaccine type			
BNT162b2	[Reference]		< 0.001
mRNA-1273	0.245	0.193, 0.297	
Inferred natural infection	0.400	0.315, 0.485	< 0.001
Race			
White	[Reference]		
Black/African American	−0.046	−0.144, 0.053	0.36
Asian	0.002	−0.064, 0.069	0.95
Hispanic/Latino	0.034	−0.072, 0.139	0.53
Other race(s)	0.106	−0.012, 0.225	0.08
Sex			
Male	[Reference]		
Female	0.059	0.007, 0.111	0.03

To account for any time-related effects of antibody levels, a much tighter window of 60 to 90 estimated days was used to visualize antibody levels among participants with reported mRNA-1273 and BNT162b2 vaccines and inferred recent natural infection or not. Participants with the mRNA-1273 vaccine still have slightly higher antibody levels, but effects are minimal given small sample sizes.

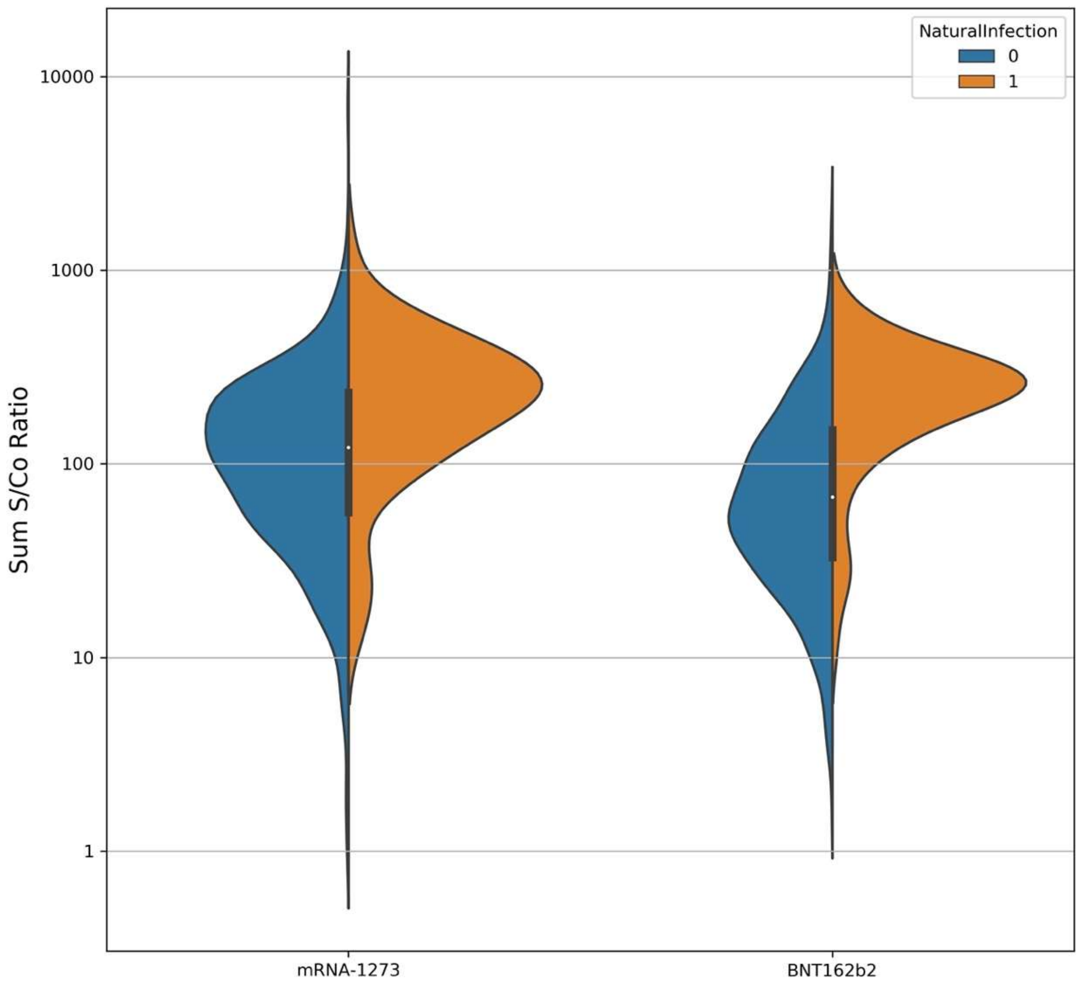

Figure S7 — Antibody Levels in mRNA-1273 and BNT162b2 (BNT162b2) Vaccine Recipients and Individuals Who Were Negative (0) (mRNA-1273 N: 38, BNT162b2 N:78) and Positive (1) (mRNA-1273 N: 5, BNT162b2 N: 18) for an Inferred SARS-CoV-2 Infection, Using a Restricted Time Window of 60–90 Estimated Days Since Reported Vaccination Date

REFERENCES

1. U.S. Census Bureau website. 2020 American Community Survey: Code Lists, Definitions, and Accuracy. https://www.census.gov/programs-surveys/acs/technical-documentation/code-lists.html. Accessed 17 December 2020.
2. U.S. Census Bureau website. American Community Survey: Sample Size and Data Quality. https://www.census.gov/acs/www/methodology/sample_size_and_data_quality/. Accessed 17 December 2020.
3. Census Reporter website for Howard County, Maryland. https://censusreporter.org/profiles/05000US24027-howard-county-md/. Accessed November 2021.
4. Sandro Galea, Melissa Tracy. Participation Rates in Epidemiologic Studies. *Annals of Epidemiology*. 2007;17 (9):643-653. https://doi.org/10.1016/j.annepidem.2007.03.013.
5. Howard County COVID-19 Dashboard. https://www.howardcountymd.gov/health/covid-19#cases-dashboard. Accessed November 2021.
6. U.S. Census Bureau QuickFacts website for Howard County, Maryland. https://www.census.gov/quickfacts/fact/table/howardcountymaryland/PST045219. Accessed November 2021.
